# Thermal Compact Modeling and Resistive Switching Analysis in Titanium Oxide-Based Memristors

**DOI:** 10.1021/acsaelm.3c01727

**Published:** 2024-02-15

**Authors:** Juan B. Roldán, Antonio Cantudo, David Maldonado, Cristina Aguilera-Pedregosa, Enrique Moreno, Timm Swoboda, Francisco Jiménez-Molinos, Yue Yuan, Kaichen Zhu, Mario Lanza, Miguel Muñoz Rojo

**Affiliations:** † Departamento de Electrónica y Tecnología de Computadores, Universidad de Granada, Facultad de Ciencias. Avenida Fuentenueva s/n, 18071 Granada, Spain; ‡ IHP-Leibniz-Institut für innovative Mikroelektronik, 15236 Frankfurt (Oder), Germany; § CEMDATIC—E.T.S.I Telecomunicación, Universidad Politécnica de Madrid (UPM), 28040 Madrid, Spain; ∥ Department of Thermal and Fluid Engineering, Faculty of Engineering Technology, University of Twente, 7500 AE Enschede, The Netherlands; ⊥ Materials Science and Engineering Program, Physical Sciences and Engineering Division, King Abdullah University of Science and Technology (KAUST), Thuwal 23955-6900, Saudi Arabia; # MIND, Department of Electronic and Biomedical Engineering, Universitat de Barcelona, Martí i Franquès 1, E-08028 Barcelona, Spain; ∇ 2D Foundry, Instituto de Ciencia de Materiales de Madrid (ICMM), CSIC, Madrid 28049, Spain

**Keywords:** memristor, resistive switching, thermal analysis, device simulation, modeling, variability analysis

## Abstract

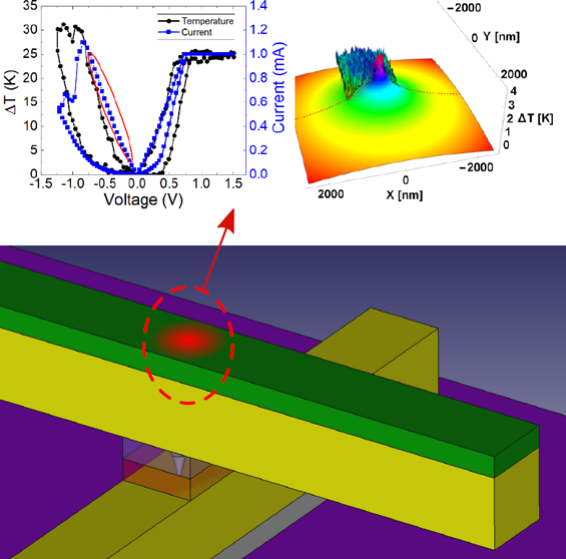

Resistive switching devices based on the Au/Ti/TiO_2_/Au stack were developed. In addition to standard electrical characterization by means of *I*–*V* curves, scanning thermal microscopy was employed to localize the hot spots on the top device surface (linked to conductive nanofilaments, CNFs) and perform in-operando tracking of temperature in such spots. In this way, electrical and thermal responses can be simultaneously recorded and related to each other. In a complementary way, a model for device simulation (based on COMSOL Multiphysics) was implemented in order to link the measured temperature to simulated device temperature maps. The data obtained were employed to calculate the thermal resistance to be used in compact models, such as the Stanford model, for circuit simulation. The thermal resistance extraction technique presented in this work is based on electrical and thermal measurements instead of being indirectly supported by a single fitting of the electrical response (using just *I*–*V* curves), as usual. Besides, the set and reset voltages were calculated from the complete *I*–*V* curve resistive switching series through different automatic numerical methods to assess the device variability. The series resistance was also obtained from experimental measurements, whose value is also incorporated into a compact model enhanced version.

## 1 Introduction

Memristors based on resistive switching (RS) are being scrutinized at academic and industrial research centers. The potential of these electron devices is outstanding at the commercial level, and different niche applications have already been put in the market.^1^ Some of these memristors change their internal resistance by means of the creation and destruction of a conductive nanofilament across an insulator layer (this layer is sandwiched between two metals, i.e., a metal–insulator–metal, MIM, structure) that shorts the metallic electrodes. These types of devices are known as resistive memories, and they are included by several companies in their technologies as nonvolatile memories (TSMC for its 40,^2^ 28,^3^ and 22 nm^4^ nodes, as well as INTEL for its 22 nm^5^ node).

When the CNF is formed, the device is in the low-resistance state (LRS); conversely, when the CNF is broken (after switching from the LRS), it is said to be in the high-resistance state (HRS). This digital operational viewpoint allows their use in memory circuits; however, if the analog perspective is considered in terms of the device conductance variation, new applications come up such as neuromorphic engineering, where these memristive devices offer in-memory computing capabilities, that lead to new architectures that can overcome the limitations of von Newmann’s bottleneck.^6^ The role of memristors within this new paradigm^7−16^ is essential to reduce energy consumption in artificial intelligence computation, since circuits based on conventional MOS transistors to implement artificial neurons and synapses are more power-inefficient. In addition, resistive memories can also be used for hardware cryptography as entropy sources to build physical unclonable functions and true random number generators.^17−19^

It is known that RS is controlled by the application of an electric field and also by the device internal temperature that is increased by Joule heating.^20−24^ In fact, the physical mechanisms behind RS are thermally activated; hence, thermal effects are key to understanding and controlling the device operation. Consequently, an accurate description of these effects is essential to build compact models for circuit simulations.^21,22,25−27^

Here, we study the RS features of devices based on Au/Ti/TiO_2_/Au stacks. We fabricate them and measure *I*–*V* curves under the ramped voltage stress (RVS) operation regime. An in-depth analysis of the experimental data is performed making use of different numerical methods to extract RS parameters. In addition, a study of the cycle-to-cycle variability^22,28,29^ is performed to understand the experimental data structure. The information on heat dissipation produced by the filament for these devices is provided in ref (30). An operando scanning thermal microscope (SThM) was used to characterize the device surface, localize the device CNFs, and extract the temperature in the hot spots. The results are contrasted with physical simulations by means of the COMSOL Multiphysics simulation tool, and the device charge conduction and temperature distributions are analyzed. Both experimental current and temperature distributions are used to tune the simulator. Finally, we go through a compact modeling stage where the Stanford model^31−34^ is adapted to fit experimental and simulation data, and essential parameters such as thermal resistance are extracted.

## 2 Device Characterization, and Electrical Measurement Setup

**Figure 1:**
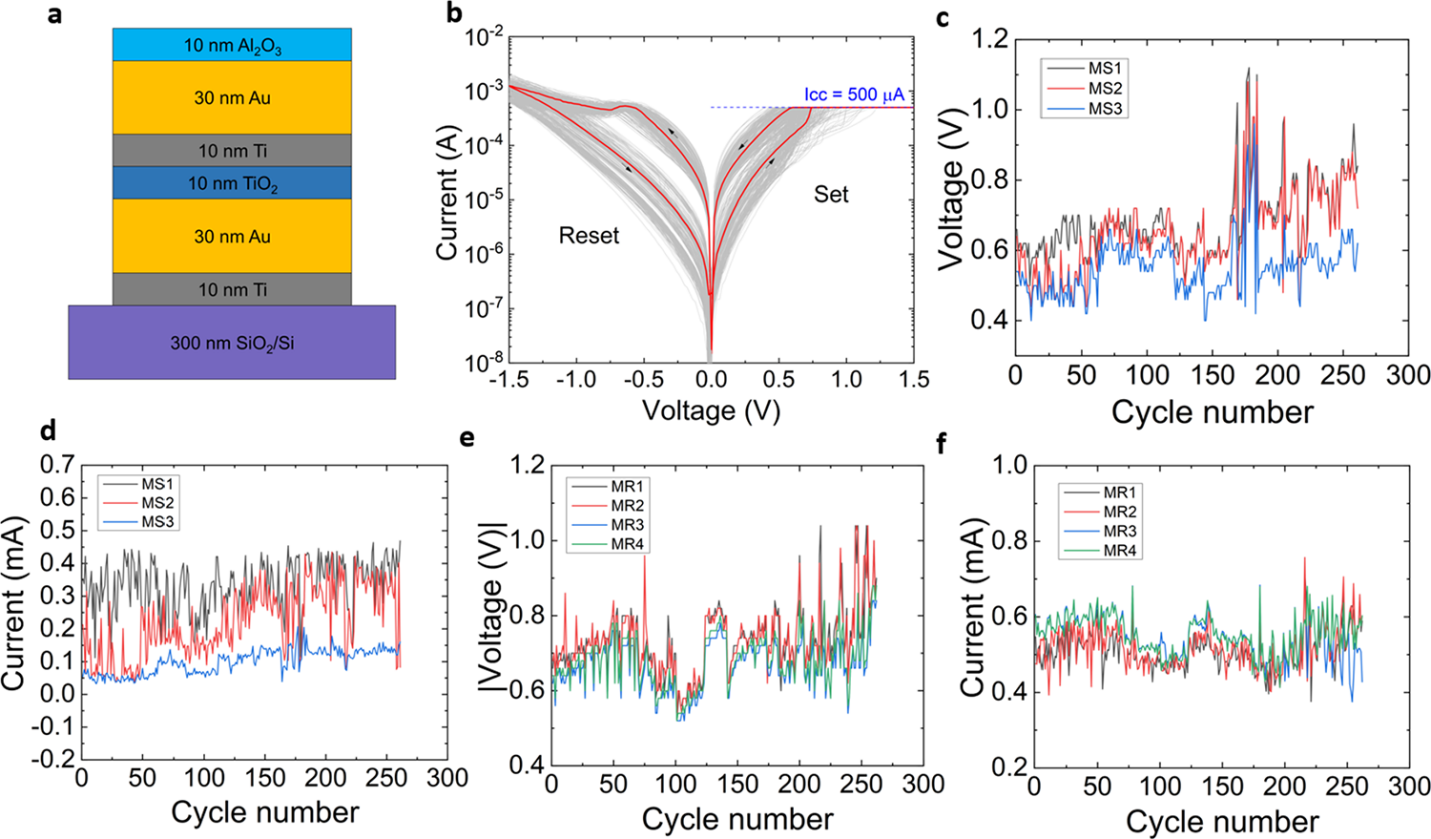
(a) Device cross-sectional schematic. (b) Experimental current vs voltage curves for 261 consecutive RS cycles (for a 2 × 2 μm device) measured for *I*_CC_ = 500 μA (the voltage ramp was 0.57 V/s). The mean of the curves measured is colored red, with arrows showing the typical hysteresis of a memristive device. (c) Set voltages vs cycle number including three different numerical techniques (MS1, MS2, and MS3) used for the set voltage extraction (see Supporting Information note 1 for the detailed explanation of the numerical procedures). (d) Set current vs cycle number corresponding to the voltages extracted in (c). (e) Reset voltages vs cycle number including four numerical techniques (MR1, MR2, MR3, and MR4) employed for the reset voltage extraction (see Supporting Information note 1). (f) Reset current vs cycle number corresponding to the voltages extracted in (e).

The cross-sectional scheme of the devices we fabricated is shown in Figure 1a. The fabrication process is explained in the Methods and Materials section. The devices were measured by means of a Keysight B1500A semiconductor parameter analyzer and a probe station (Karl Suss PSM6). A B1511B medium power source measurement unit (MPSMU) was used for the quasi-static ramped voltage stress measurements. The bottom electrode was grounded, and the input voltage signal was applied to the top electrode. A ramped voltage stress (RVS) operation regime was employed for the device characterization. The *I*–*V* curves measured are shown in Figure 1b, including more than 250 cycles. An average curve is highlighted in red to let the reader see the *I*–*V* curve shape, which looks similar to that of valence change memories based on HfO_2_.^21,28^

The set and reset voltages and currents (current levels at the *I*–*V* point determined by the corresponding set and reset voltages) have been obtained by making use of different techniques (the numerical details of each procedure are described in the Supporting Information note 1). The set (reset) voltages and currents extracted from the experiments are plotted in Figure 1c,d (e and f). For the reset voltage, a higher variability is shown for the MR1 and MR2 techniques.

## 3 Device Thermal Characterization (Scanning Thermal Microscopy)

The thermal characterization of the devices was carried out with a scanning thermal microscope (SThM), as described in ref (30) and in the Supporting Information note 2. For that purpose, we used an Asylum MFP-3D atomic force microscope with a SThM add-on from Bruker. For sensing the devices, we used thermoresistive GLA-1 probes from Bruker. The electrical resistance of these probes varies with temperature, and it is connected electrically to a Wheatstone bridge to sense these variations during surface scans. We operated the SThM in sensing mode using a probe power of 19 μW, which, as determined in a prior study, is optimum for measuring under sensing conditions.^35^

In that mode, we kept the probe at a low self-heating temperature while achieving a high temperature sensitivity. For the conversion of the SThM Wheatstone bridge voltage signal (mV) into temperature, we calibrated the system as described in ref (35).

A Keithley 4200 A-SCS parameter analyzer was used in combination with the SThM to study the heat dissipation in the memristor devices when applying *I*–*V* curves in between the two contact pads.^30^ In these measurements, we studied the TiO_2_-based RRAM devices with a cross-point area size of 2 × 2 μm^2^ and a cross-section structure, as described in the previous section. In order to verify the cyclability of our devices, we performed more than 10 RS cycles before doing the SThM scans. During the cycling, we limited the current using a compliance of 1 mA for the set process.

**Figure 2:**
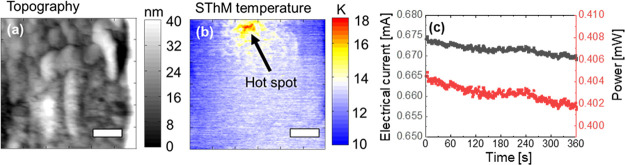
(a) Steady-state topography map of the cross-point of a TiO_2_-based RRAM device with an area size of 2 × 2 μm^2^ in its LRS. (b) SThM temperature map corresponding to the scan in (a). The map shows a hot spot with an elevated temperature induced by the electrical current flowing through the CNF (scale bar corresponds to 200 nm). (c) Electrical current and power are applied to the device as functions of the scan time.

Once we verified the cyclability, we carried out SThM steady-state measurements at the cross-point area while applying a constant current to identify the heat dissipation at the surface. Figure 2a,b shows the topography and the temperature map of the cross-point area of a biased 2 × 2 μm^2^ device in its low resistive state (LRS). The applied current and electrical power during the steady-state scan are plotted in Figure 2c. In the LRS, the device is set, and the electrical current flows through the formed and confined CNF. The heat dissipation of the generated filament Joule heating is then localized as a hot spot at the surface, as illustrated in Figure 2b. At this point it is worth noting that the heat dissipation between CNF and the surface varies depending on the device material selection and thickness, and hence, it must be carefully analyzed.^36^

Afterward, we kept the SThM probe static at the initial hot spot position, as localized by the steady-state measurements. Then, in order to correlate the electrical signal with temperature, in-operando, we ran *I*–*V* cycles while tracking the SThM signal at the hot spot position. The results of these measurements are presented in the Results and Discussion.

## 4 Device Simulation

**Figure 3:**
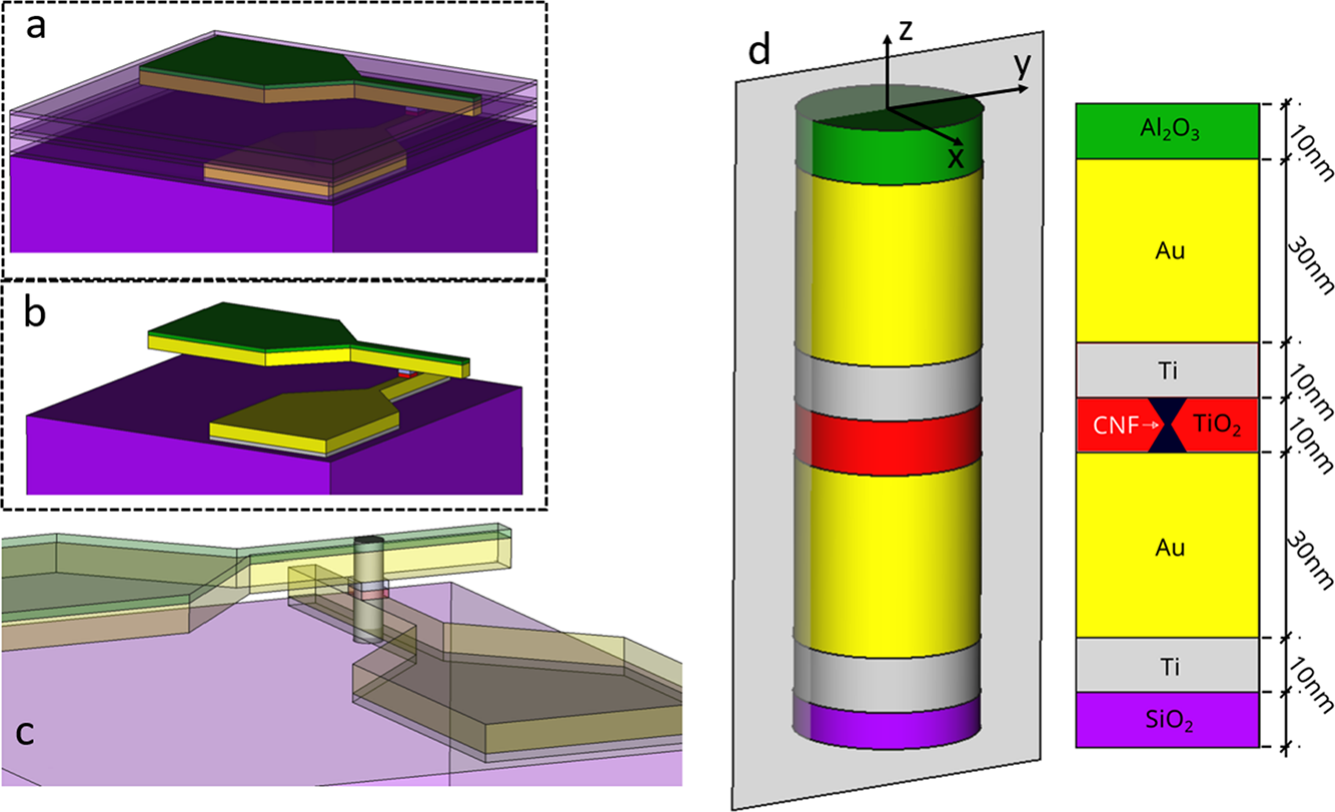
(a) Device fabrication scheme: titanium oxide is employed as a dielectric and bilayers of Au/Ti as electrodes. (b) Device view in the crossbar structure. (c) Cylindrical simulation domain containing the main device layers (zoomed-in view from b). (d) 3D longitudinal device cross section where the revolution geometry that constitutes the COMSOL simulation domain is shown.

For device simulation, we employed the COMSOL Multiphysics simulation tool. The physical model developed is in line with the one reported in ref (30) although we have included improvements to correctly describe the internal device thermal behavior. In this respect, we considered the simulation structure shown in Figure 3.

It consists of a revolution geometry that forms the simulation domain (Figure 3c,d). The layer structure is given in Figure 3d, in line with previous approaches.^30^ We assume a CNF with an hourglass shape, with the maximum and minimum radius of 6 and 2.9 nm, respectively.

Some of the material properties are given in Table 1, while others are extracted from ref (30). The CNF electrical conductivity is assumed to be 2.85 × 10^5^ (Ωm)^−1^ at *T* = 300 K. Since the conductive filament is fully formed and shorts the electrodes (the filament is in contact with both the bottom and top electrodes), heat transport is more efficient through the filament than through the surrounding oxide; in this respect, interfacial oxides formed between Ti and the dielectric are not expected to influence much when the device operates in the low-resistance state.

**Table 1:** Thermal Conductivities of the Materials Employed in the Simulation

material	*k* [W/(m K)]	ref
Ti	8.2	(37)
Au	317.15	
TiO_2_	0.8	(38)
SiO_2_	1.4	
Al_2_O_3_	3.45	(24)
conductive nanofilament	18	(20,27)

In the reset process experimental data (the CNF is fully formed at the beginning of the reset process), the current versus voltage relationship is not completely linear. Therefore, in order to implement this nonlinearity, we include a constriction (described with the quantum point contact, QPC, model^39−41^) in series with the ohmic CNF, as it has been implemented for the modeling of other devices.^20,42^ In this manner, the external applied voltage (*V*_RRAM_) is the sum of the voltage in the CNF (*V*_CNF_) and the voltage at the constriction (*V*_CTR_), so that *V*_RRAM_ = *V*_CNF_ + *V*_CTR_. The QPC physics has been described previously,^39−41^ and the current in the constriction that quantizes the electron energy in the CNF transverse direction is given in eq 1.
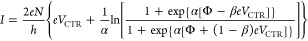
1Landauer’s formalism for 1D quantum conductors and the zero-temperature limit were employed^39,40^ to obtain eq 1, where Φ is the potential barrier height measured with respect to the Fermi level, α is a parameter linked to the potential barrier thickness at the Fermi level, *V*_CTR_ is the voltage which is assumed to drop at both ends of the CF constriction (in a fraction of β and (1−β) at each extreme, as suggested in ref (43)) and *N* is the number of channels. The QPC parameters employed in the fitting of the experimental data of our devices are the following: β = 0.99, ∝ = 14 eV^–1^, *N* = 242, and Φ = 0.134 eV.

## 5 Results and Discussion

### 5.1 Parameter Extraction

**Figure 4:**
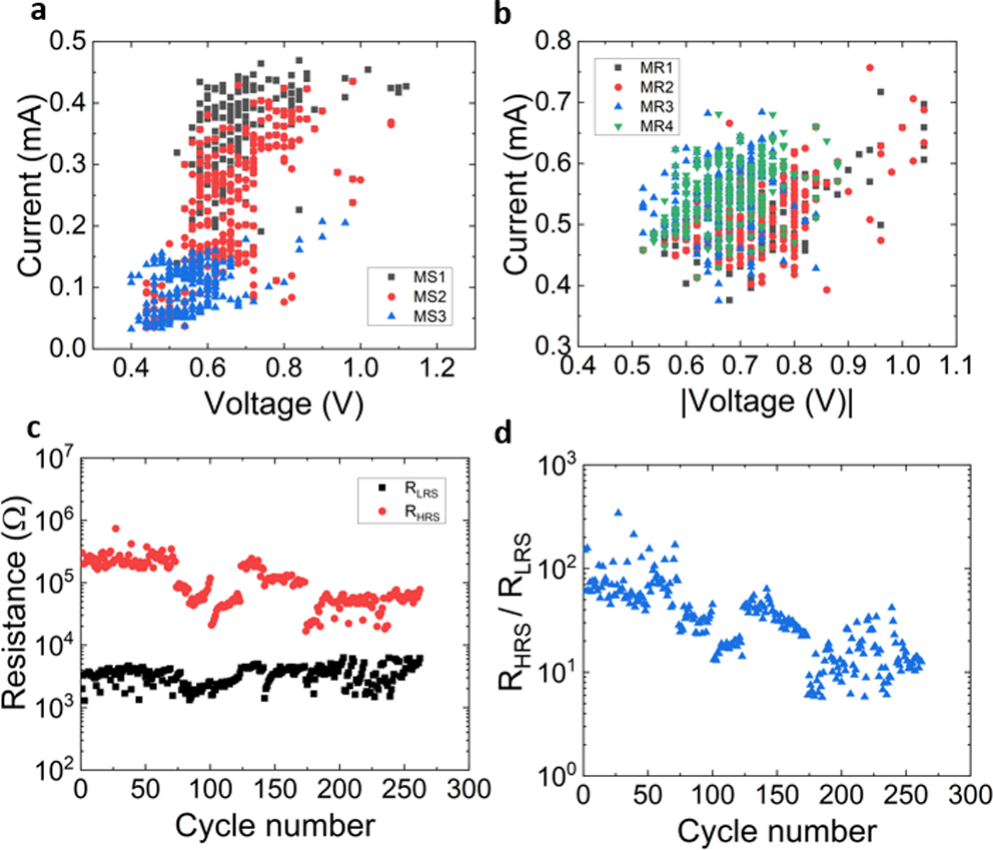
(a) Experimental *I*_set_ vs *V*_set_ extracted, employing methods MS1, MS2, and MS3. (b) Experimental *I*_reset_ vs *V*_reset_ extracted, switching voltage parameters, employing methods MR1, MR2, MR3, and MR4. (c) LRS and HRS resistances (read at 0.2 V) vs cycle number for all the measured RS series. (d) HRS/LRS resistance ratio vs cycle number calculated with data from (c).

As highlighted above, the set and reset voltage extraction procedures are explained in the Supporting Information 1 and Figure S1. If the set and reset currents are plotted versus the corresponding voltages (Figure 4a,b), we see that the reset parameters present less variability, which is easily seen in the cumulative distribution functions plotted in Figure S2a–d.

It is observed that *R*_HRS_/*R*_LRS_ is high enough to let the use of these devices feasible for nonvolatile memory applications, and the values found for *R*_HRS_ and *R*_LRS_ are coherent in comparison to other memristive devices.^28^

### 5.2 RS Parameter Statistics

We performed a statistical analysis to untangle the structure of the data obtained in previous sections. To do so, we obtained the mean values, standard deviations, and coefficients of variation (CV, calculated as σ/μ, where σ stands for the standard deviation and μ for the mean) for each RS parameter; see Tables 2 and 3 for the set (reset) parameters. In general, if the cycle-to-cycle variability is low (i.e., CV of *V*_set_ < 2%), the devices could be used for information storage,^44^ computation,^6^ or transmission;^45^ if the variability is high (CV of *V*_set_ > 20%), the devices could rather be employed for data encryption as entropy source for true random number generators^18^ or physical unclonable functions.^46^

**Table 2:** Statistical Study of the Extracted Set of RS Parameters for the Different Extraction Methodologies^a^

parameter	mean (μ)	standard deviation (σ)	coefficient of variation (σ/μ)
*V*_MS1_	0.68789 V	0.10654 V	0.15488
*V*_MS2_	0.64782 V	0.11663 V	0.18004
*V*_MS3_	0.55103 V	0.08048 V	**0.14606**
*I*_MS1_	3.32701 × 10^–4^ A	7.83586 × 10^–5^ A	**0.23552**
*I*_MS2_	2.2049 × 10^–4^ A	1.0575 × 10^–4^ A	0.47961
*I*_MS3_	9.95904 × 10^–5^ A	3.76061 × 10^–5^ A	0.37761

a The minimum CV values are highlighted.

**Table 3:** Statistical Study of the Reset RS Parameters for the Different Extraction Methodologies^a^

parameter	mean (μ)	standard deviation (σ)	coefficient of variation (σ/μ)
*V*_MR1_	0.73267 V	0.08869 V	0.12105
*V*_MR2_	0.73084 V	0.08786 V	0.12022
*V*_MR3_	0.6658 V	0.06492 V	**0.09751**
*V*_MR4_	0.68092 V	0.07063 V	0.10373
*I*_MR1_	5.08099 × 10^–4^ A	5.09536 × 10^–5^ A	0.10028
*I*_MR2_	5.17962 × 10^–4^ A	5.46663 × 10^–5^ A	0.10554
*I*_MR3_	5.47164 × 10^–4^ A	5.32173 × 10^–5^ A	0.09726
*I*_MR4_	5.53214 × 10^–4^ A	5.09291 × 10^–5^ A	**0.09206**

a The minimum CV values are highlighted.

As known, a lower CV indicates lower variability. The results demonstrate, as expected, that the CV depends on the extraction methodology. This is a key result that makes it clear that the extraction numerical procedure should be clarified in the literature. In particular, MS3 presents the lowest value for *V*_set_, whereas MS1 is for *I*_set_. MR3 marks the minimum variability for *V*_reset_, while MR4 is for *I*_reset_.

In filamentary-based memristive devices, the reset process normally exhibits higher variability than the set;^1,47,48^ however, in our devices, the behavior is different. Therefore, a different role of thermal and electric field effects is expected to lead to the homogenization of the *V*_reset_ distribution.

**Figure 5:**
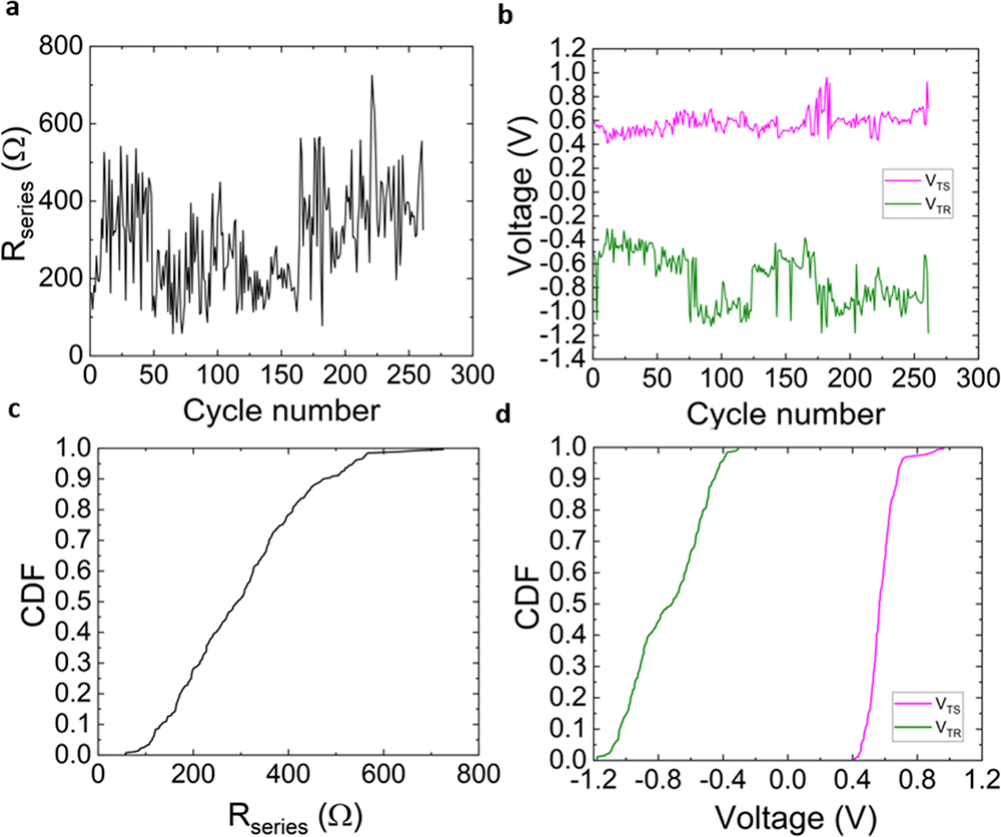
(a) Series resistance computed for the complete RS series as a function of the cycle number for the data analyzed. (b) Set transition voltage (*V*_TS_) and reset transition voltage (*V*_TR_) plotted against the cycle number for the complete RS series of the data under study. Cumulative distribution functions for the studied parameters in the whole RS series: (c) series resistance, (d) transition voltages for the set (*V*_TS_) and for the reset processes (*V*_TR_).

We have also extracted the series resistance of our devices following a previously published extraction technique;^49,50^ see Figure 5a. In addition, the reset and set transition voltages were extracted^50^ (they stand for the reset and set voltages, once the effects of the series resistance have been extracted from the original current vs voltage curve, what is known as the normalized *I*–*V* curve); see Figure 5b. The CDFs for these parameters are listed in Figure 5c,d. It is observed, as also reported in ref (50) for the HfO_2_ technology, that the absolute values of the reset and set transition voltages are much more similar than the original set and reset voltages. However, the series resistances extracted are higher than that in the HfO_2_ devices analyzed in ref (50).

### 5.3 Device Physical Simulation

**Figure 6:**
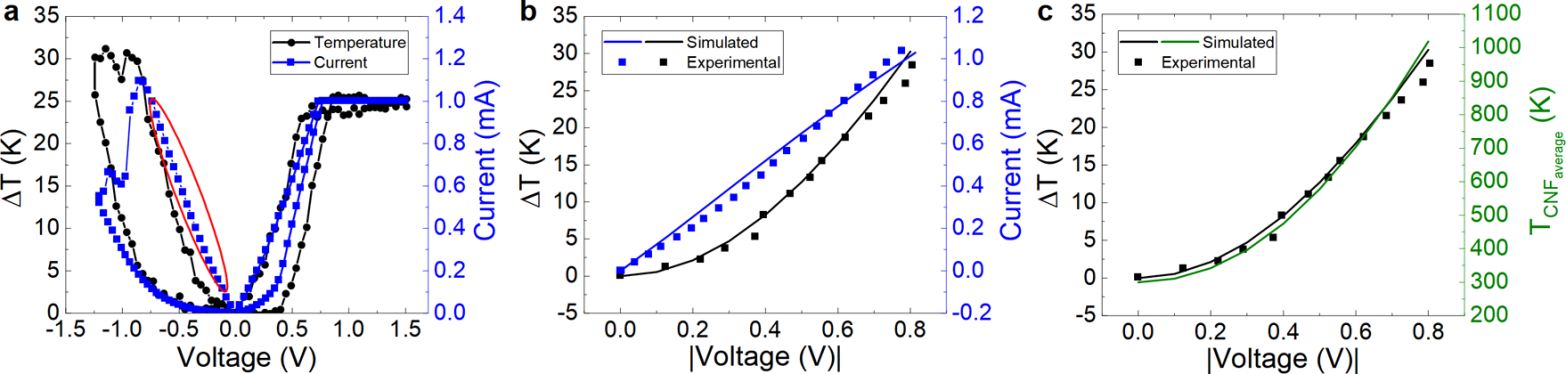
(a) Experimental current vs voltage (in blue) and temperature increment (in black, obtained with SThM) on top of the device outer Al_2_O_3_ layer vs voltage (the current curve corresponds to the temperature increment curve; i.e., in-operando measurements), (b) simulated (straight lines) and experimental data (in dots) for the reset process (the experimental data correspond to the curve highlighted in the red ellipse in (a). (c) Simulated (straight lines) and experimental (black dots) temperature increment on top of the Al_2_O_3_ layer and simulated CNF average temperature along the reset process *I*–*V* curve (this latter curve is needed for the compact modeling process).

We have made use of the simulation approach described in Section 4. The reset process was simulated by assuming that the CNF is fully formed at the beginning of the simulation. We employ the first part of the reset *I*–*V* curve (see Figure 6a), prior to the reset event, in order to fit the experimental current and also the temperature in certain parts of the device. In Figure 6a, we show the current versus voltage (blue data) and temperature increment (black data) on top of the Al_2_O_3_ layer measured with the SThM technique (in an in-operando manner). In particular, we simulated the reset curve (highlighted with a red ellipse); in this case, we use a fully formed hourglass-shaped CNF, as shown in Figure 3. Simulation and in-operando experimental data for the reset process highlighted in Figure 6a are shown in Figure 6b showing a reasonably good fit, taking into account the complexity of simultaneously reproducing temperature and current data.

Once the COMSOL simulation model was tuned, we calculated the average CNF temperature. This temperature is employed in compact models since just one temperature is usually assumed in the device for each bias point. In this respect, simplified thermal models can be built to describe the devices in the circuit simulation approach.^25^

**Figure 7:**
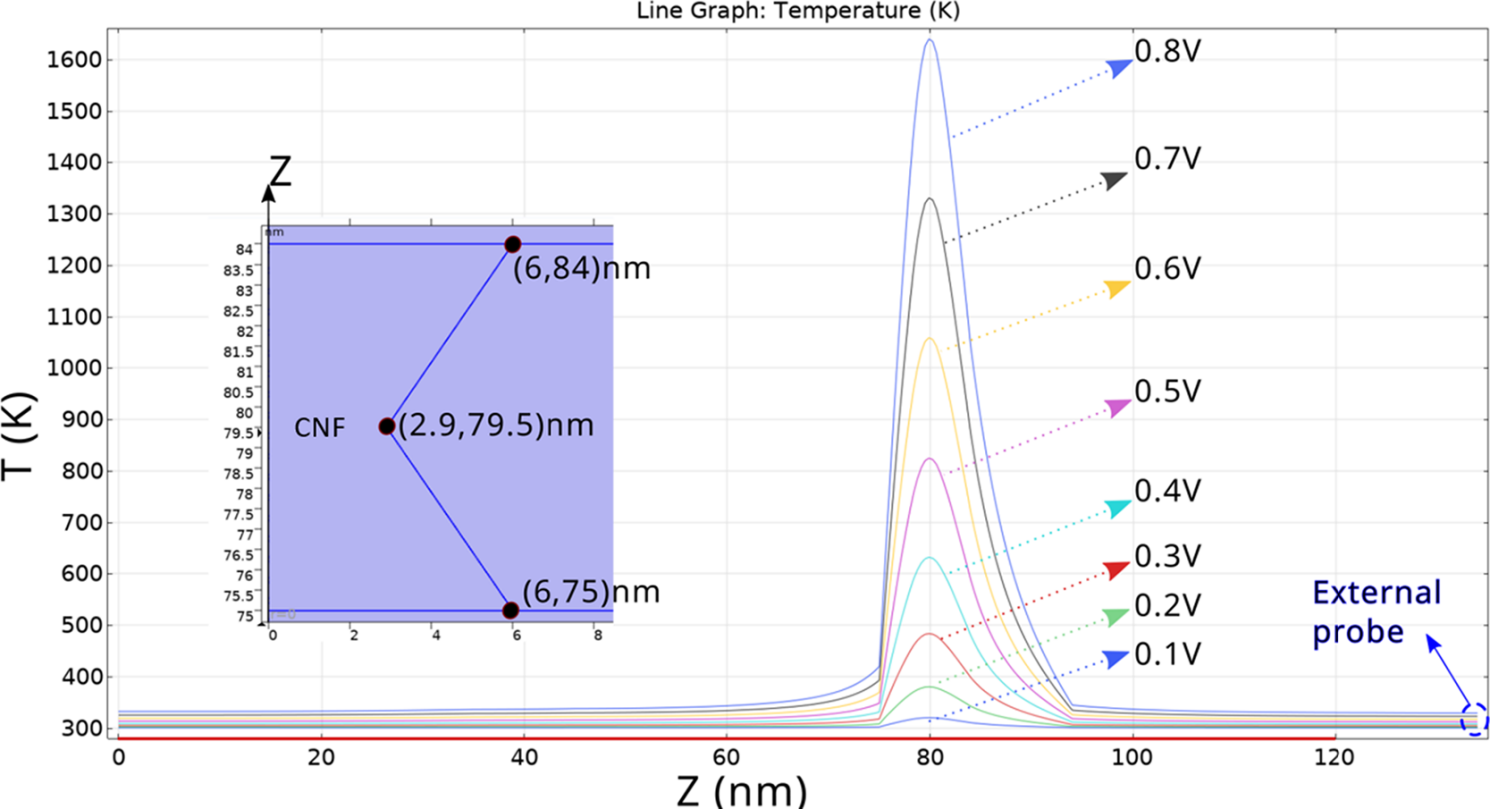
Temperature at the center of the conductive filament vs *z* coordinate (vertical device coordinate) in our simulation domain for different external voltages. CNF position and the *z*-axis orientation are seen in the inset. The higher temperature is obtained at the CNF narrowing (at the center of the hourglass structure).

A closer look at the CNF temperature allows us to detail the thermal distribution along its length (Figure 7). See the temperature peaks at the CNF narrowing, as it should be since Joule heating increases at this point because of the current line concentration. Notice also the fast temperature decrease outside the filament region, mostly at the Au side.

**Figure 8:**
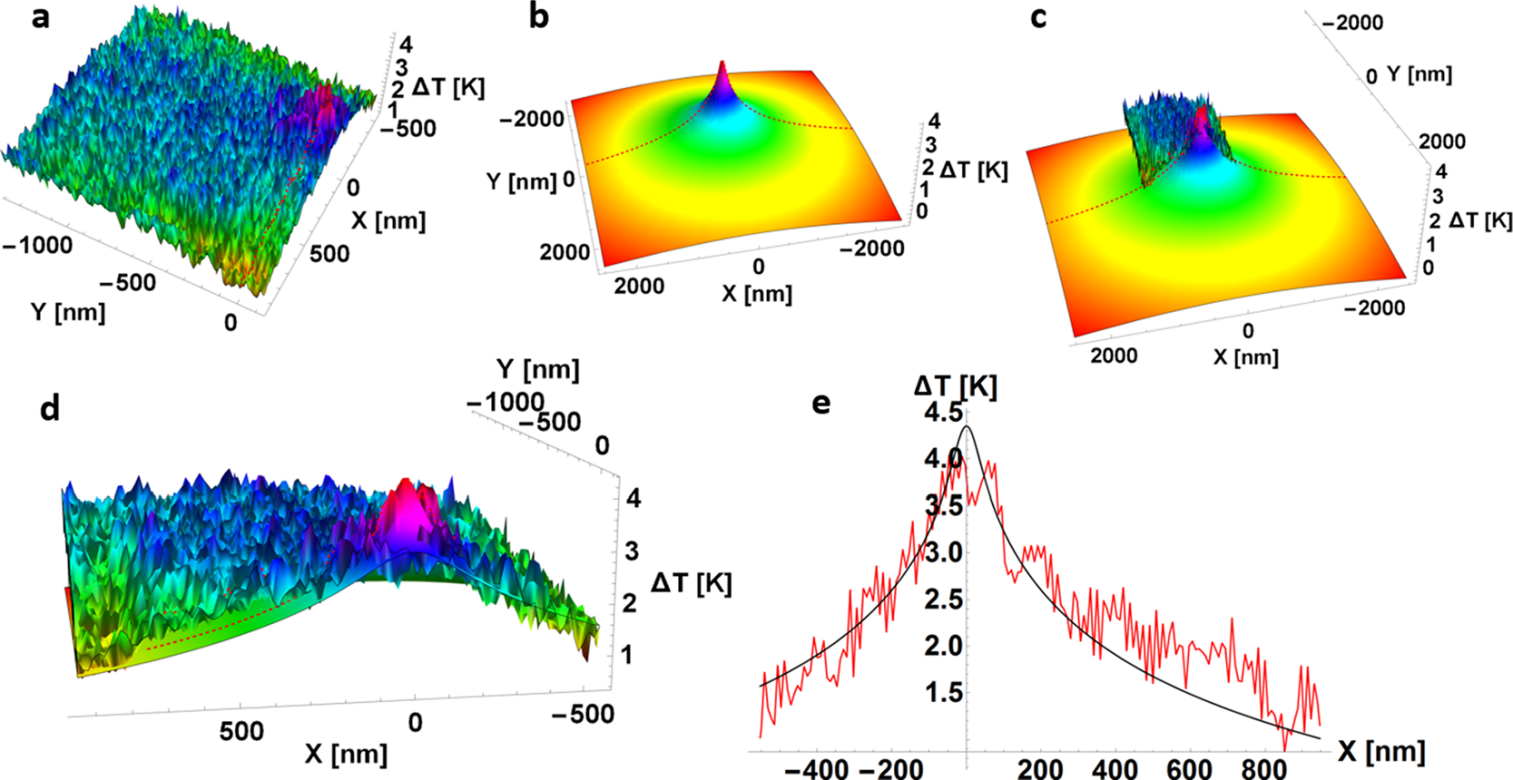
(a) Three-dimensional experimental plot (temperature increase with respect to room temperature) on top of the Al_2_O_3_ layer for *V* = 0.3 V. (b) Corresponding COMSOL simulation (after tuning) for the device scheme in Figure 3 and *V* = 0.3 V. (c) Comparison between the simulated and measured distributions; (d) panel; (c) zoomed-in view. (e) Good fit is obtained for the different cuts in the experimental/simulated distributions at different simulation domain orientations; this one is taken along the *x-*axis.

It is worth also mentioning the good fit obtained by comparing the simulated and measured distributions of temperature increase at the device surface (Figure 8). A good result is obtained throughout the simulation domain top surface. These results suggest the correctness of the model proposed.

### 5.4 Compact Modeling

**Figure 9:**
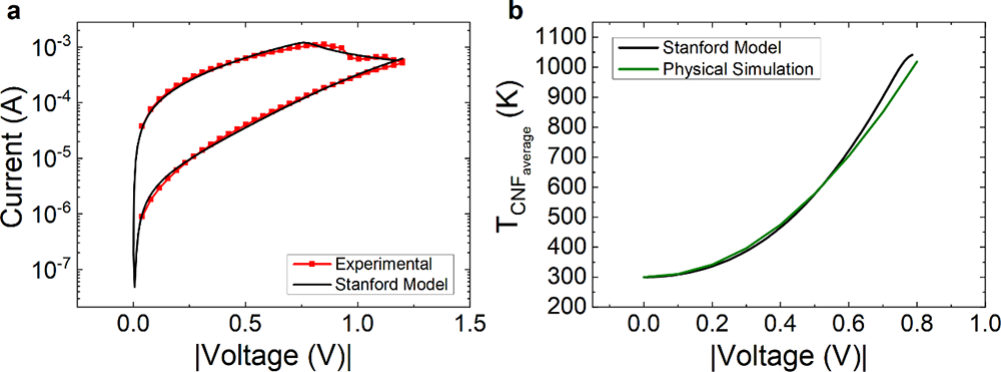
(a) Current vs voltage for a reset process. Experimental data are shown in red symbols, and those obtained with the Stanford model are shown in lines. (b) Average temperature in the CNF obtained with the COMSOL simulation tool (green line) and device temperature obtained with the Stanford model (black line).

For the compact modeling approach, the Stanford model^31−34^ is employed. This widely known model uses an equivalent single RC electrical network, driven by a current source, for representing the thermal behavior (thermal resistance and capacitance) and the Joule heating, respectively. We were able to fit the experimental reset curve highlighted in Figure 6, as shown in Figure 9.

We employed the model parameters described in Table 4. In particular, for the determination of the thermal resistance, we used the COMSOL simulated data of Figure 9b (average CNF temperature). At this point, we highlight the fact that, when fitting *I*–*V* curves in developing memristor models, we usually do not have the information given in Figure 9b, which is connected to the SThM measurements, and therefore, the thermal resistance (*R*_th_) cannot be accurately determined. In our case (see Table 4), the value is 1.2 × 10^6^ K/W, which is in line with those reported in refs (25, 31).

**Table 4:** Stanford Model Parameters Used in Figure 9^a^

symbol	value	symbol	value
*t*_ox_	10 nm	*E*_a_	1.05 eV
*I*_0_	50 mA	*E*_m_	3.25 eV
*V*_0_	0.2 V	*g*_max_	6.3 nm
*g*_0_	0.7 nm	*g*_ini_	5.2 nm
β	10.5 (reset)	*T*_0_	300 K
*v*_0_	5 × 10^6^ m/s	*T*_crit_	450 K
γ_0_	20	*R*_series_	220 Ω
α	1.1 (reset)	*R*_th_	1.2 × 10^6^ K/W

a *R*_series_ is a series resistance added to the nonlinear current source of the Stanford model.^50^

The analytical expression to determine the thermal resistance is given in eq 2. In our case, accounting for the low-frequency RVS operation measurements performed (this implies steady-state operation), the transient part of the modified heat equation can be excluded for the usual thermal capacitance values found for resistive memories.^25^
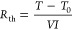
2We also used a series resistance in the compact modeling approach, following a previous work,^50^ where the inclusion of this parameter and the effects on the simulation are described.

## 6 Conclusions

An experimental characterization of resistive switching in Au/Ti/TiO_2_/Au devices has been presented, including data obtained with surface scanning thermal microscopy. Macroscopic simulations with COMSOL Multiphysics are performed in order to link the electrical and thermal measurements. Quantum effects are considered in the simulations performed. The experimental and simulated data are used together to calibrate a compact model (an enhanced version of the widely used Stanford model). In this way, temperature in-operando measurements of hot spots on the top device surface, linked to the position of CNFs, and simulations allow us to describe the CNF internal temperature for modeling. A good agreement between simulated and experimental data is achieved, for both the current and CNF temperature. The average CNF temperature is employed to extract the device thermal resistance. This parameter is indirectly determined in compact modeling by fitting the electrical characteristics (*I*–*V* curves). On the contrary, the procedure presented in this work permits a direct estimation, linked to thermal measurements. The device characterization is completed by the extraction of set/reset voltages and series resistance from the experimental *I*–*V* curves. The latter parameter is also incorporated in the compact model.

## 7 Methods and Materials

### 7.1 Device Fabrication

The devices were built on a 300 nm SiO_2_/Si substrate. A 30 nm gold layer was deposited as the bottom electrode on top of a Ti adhesion layer with a thickness of 10 nm grown by e-beam evaporation. For the dielectric, a 10 nm thick TiO_2_ layer was grown by means of atomic layer deposition (ALD). The top electrode was deposited in a similar way to the bottom electrode, including the Ti layer. For electrical isolation, an Al_2_O_3_ capping layer of 10 nm was grown by ALD. The device structure consists of a cross-point structure with contact pad sizes of 100 × 100 μm^2^. The devices were built making use of two different cross-point area sizes (2 × 2 and 5 × 5 μm^2^).^30^ The use of Au as an electrode material is not unusual; it has been employed in different academic studies^51−54^ and also in the context of the industry.^55,56^

## Data Availability

The data sets generated and/or analyzed during the current study are available from the corresponding author on reasonable request.

## Supporting Information Available

Numerical techniques employed in the extraction of the set and reset voltages; cumulative distribution functions of the set and reset voltages and currents; and thermal measurement techniques, features, resolution, and limitations (PDF)
