# Supplementary material for: Thermal Compact Modeling and Resistive Switching Analysis in Titanium Oxide-Based Memristors
Source: ACS Appl Electron Mater. 2024 Feb 15;6(2):1424–33. doi: 10.1021/acsaelm.3c01727 (PMC10903745; doi:10.1021/acsaelm.3c01727)
Supplement: Supplementary file 1 — el3c01727_si_001.pdf [file el3c01727_si_001.pdf]

SUPPORTING INFORMATION

Thermal compact modeling and resistive switching analysis in titanium oxide based memristors

Juan B. Roldán[1*], Antonio Cantudo[1], David Maldonado[1,2], Cristina Aguilera-Pedregosa[1], Enrique Moreno[3], Timm Swoboda[4], Francisco Jiménez-Molinos[1], Yue Yuan[5], Kaichen Zhu[6], Mario Lanza[5], Miguel Muñoz Rojo[4,7]

[1]Departamento de Electrónica y Tecnología de Computadores. Universidad de Granada. Facultad de Ciencias. Avenida Fuentenueva s/n, 18071 Granada, Spain.
[2]IHP-Leibniz-Institut für innovative Mikroelektronik, 15236 Frankfurt (Oder), Germany
[3]CEMDATIC - E.T.S.I Telecomunicación, Universidad Politécnica de Madrid (UPM), 28040 Madrid, Spain
[4]Department of Thermal and Fluid Engineering, Faculty of Engineering Technology, University of Twente, 7500 AE Enschede, The Netherlands
[5]Materials Science and Engineering Program, Physical Sciences and Engineering Division, King Abdullah University of Science and Technology (KAUST), Thuwal 23955-6900, Saudi Arabia
[6]MIND, Department of Electronic and Biomedical Engineering, Universitat de Barcelona, Martí i Franquès 1, E-08028 Barcelona, Spain
[7]2D Foundry, Instituto de Ciencia de Materiales de Madrid (ICMM), CSIC, Madrid 28049, Spain

*Corresponding Author Email: jroldan@ugr.es

Supplementary note 1

The numerical techniques employed in the extraction of the set and reset voltages are described below.

1.-V_{set} extraction algorithm

MS1 - Determination of the current derivative maximum.

The first method to determine the set voltage consists of finding the maximum value of the numerical derivative [1]. The determination of the numerical derivative is not an easy issue due to measurement fluctuations among other considerations; for this reason, to consider the key operation region, the I-V curve has been swept starting from 3% of the voltage range to 70%; this is, from 0.15V to 1.05V. In our case a 5-point numerical calculus for the derivative was performed, see Figure S1a; although, more elaborate techniques based on advanced approximation theory and numerical derivation could be included if needed [2, 3, 4].

MS2 - Higher current increase in two consecutive points.

Another conventional methodology consists of detecting the maximum current increase in two consecutive points evaluating all the I-V curves ($m_i = I_{i+1}/I_i$ where $V_{set} = V_j$ if $m_j = \max\{ m_i \}$). The first part of the curve is avoided because the current increase between points might be high, and this would mask the extraction process; in particular, the I-V curve has been analyzed in the [0.44 V, 1.5 V] interval, see Figure S1b.

MS3 - Maximum separation from a straight line that joins the end points in a set curve.

A different approach is based on the determination of the maximum separation between the measured I-V curve and a theoretical straight line that connects the first point in the experimental curve to the first point in the region where the current maximum is reached (see Figure S1c, where it is plotted in dashed line). We pursue the determination of the set I-V curve knee (Figure S1c). The farthest point from the dashed line to the measured I-V curve defines V_{set} [1].

2.-V_{reset} extraction techniques

MR1 - Determination of the current derivative minimum

The procedure is based on the identification of the minimum value of the current derivative. This approach is somewhat related to the first method for the set voltage, as the numerical derivative is calculated in a similar way [1]. The minimum peak of the current derivative can be easily identified numerically, see Figure S1d.

MR2 - Higher current decrease in two consecutive points.

Another conventional method involves identifying the higher current decrease (m) between two adjacent points in the I-V curve ($m_i=I_{i+1}/I_i$, where $V_{reset}=V_j$ if $m_j = \min\{ m_i \}$). To minimize potential errors, when analyzing the initial portion of the curve, where the current increase between points is high, we evaluated the I-V cycle from 0.3 V to 1 V, as shown in Figure S1e.

(MR3): Determination of the current maximum

Another approach involves calculating the maximum current, as illustrated in Figure S1f. This procedure may encounter issues related to the current maximum's location which depends on the compliance current. In cases, if the current compliance is sufficiently high, the maximum current could correspond to the last measured current in the curve. This issue should be clarified to employ this procedure.

(MR4): First point with a decreasing trend in the current curve

This approach works well [5], although it is much more sensitive to RS current jumps than previous ones. The numerical derivative is calculated (see Figure S1g) to find the first point with a negative value (the reset point).

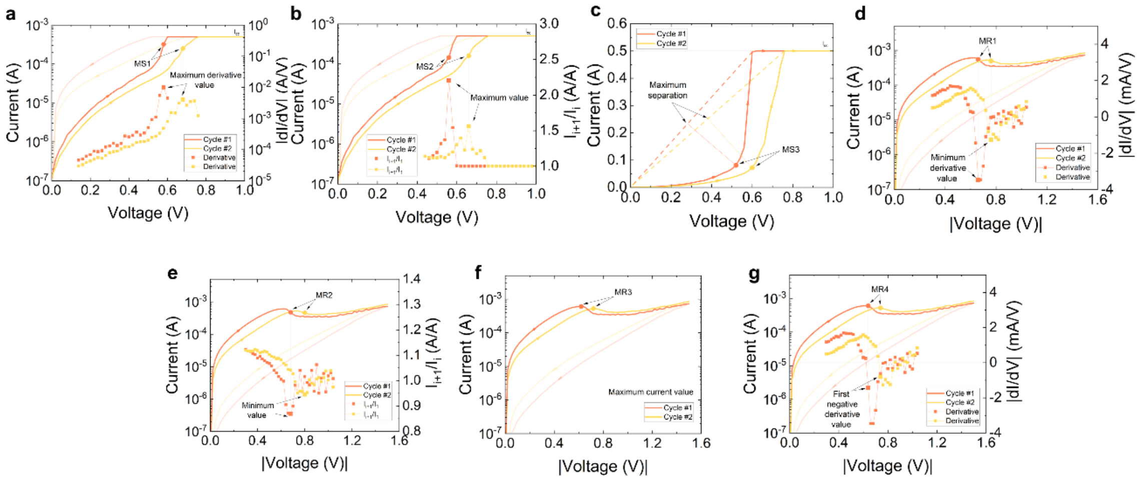

Figure S1. a Experimental set current (orange and yellow lines) and first derivatives (orange and yellow symbols) versus voltage for two different cycles. The set point is established (orange and yellow squares) at the current derivative maximum. **b** Experimental set current (orange and yellow lines) versus voltage for two example cycles. This new methodology seeks for the maximum current increase in two consecutive points (orange and yellow symbols) which determines the set point. **c** Experimental set current (orange and yellow lines) versus voltage for two example cycles. This technique uses a straight line (dashed) to join the point where the compliance current is reached and the first measured current, and the maximum distance marks the set voltage. **d** Experimental reset current (orange and yellow lines) and first current derivatives (orange and yellow symbols) versus voltage for two different cycles. The reset point is established by determining the minimum current derivative. **e** Experimental reset current (orange and yellow lines) versus voltage for two example cycles. This technique seeks for the maximum current decrease in two consecutive points which determines the reset point. **f** Experimental reset current (orange and yellow lines) versus voltage curves for two example cycles. The maximum current value is established as the reset point. **g** Experimental reset current (orange and yellow lines) and first current derivatives (symbols) versus voltage curves. The first point with negative value of the derivative was established as the reset.

The V_{set}, I_{set}, V_{reset} and I_{reset} cumulative distribution functions (CDFs) have also been calculated and plotted (Figures S2a, S2b). As can be seen the set curve knee determination (MS3) provides lower set voltages and currents than the maximum current derivative method (MS1) and the current increase in two consecutive points (MS2). For the V_{reset} (Figure S2c), the results are similar for both MR1 and MR2; for I_{reset} (Figure S2d), the maximum current method (MR3) and the one that accounts for the first point with negative derivative (MR4) provide higher I_{reset}. It is important to highlight that obtaining the reset parameters can be a challenging due to variations in the I-V curve shapes. In certain cases, as observed in these devices, the I-V curves exhibited a current increase after the reset voltage, resulting in inaccurate parameter determination. Therefore, the interval where the numerical search of the reset voltage has to be restricted.

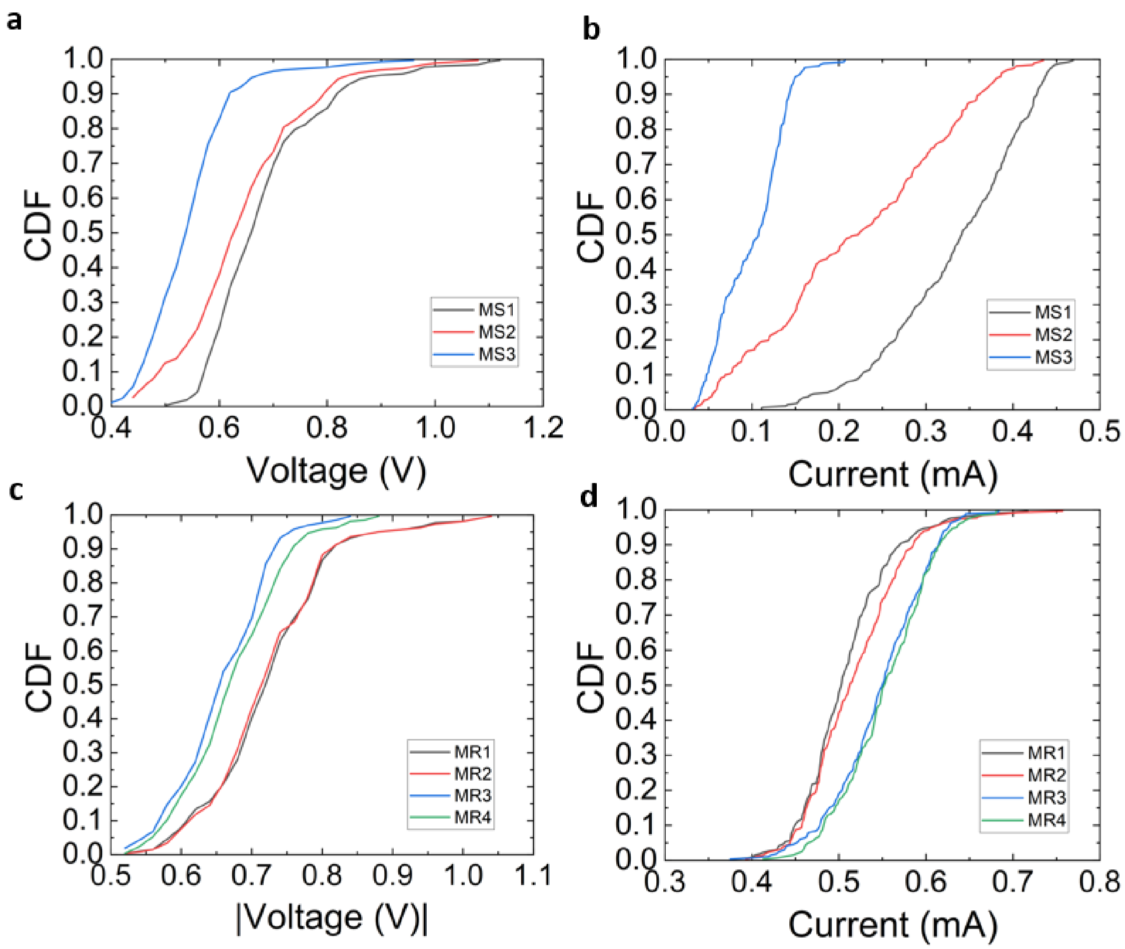

Figure S2 a V_{set} cumulative distribution functions calculated by means of methods MS1, MS2 and MS3, **b** I_{set} cumulative distribution functions calculated using methods MS1, MS2 and MS3. **c** V_{reset} cumulative distribution functions calculated by means of methods MR1, MR2, MR3 and MR4, **d** I_{reset} cumulative distribution functions calculated using methods MR1, MR2, MR3 and MR4.

Supplementary note 2

1. SThM measurements and temperature conversion

For the characterization of the surface temperature, we scanned the heated RRAM devices with a scanning thermal microscope (SThM). We utilized thermoresistive probes which are connected to an external Wheatstone bridge. While scanning the surface, we applied an electrical current to the Wheatstone bridge to heat up the probe. Changes in the electrical resistance of thermoresistive probes are connected to variations in their temperature. By that means we can sense differences in temperature due to changes of the electrical signal of the bridge. More details on the principle of these measurements can be found in previous studies [6, 7].

The conversion of the temperature maps was achieved based on the calibration approach presented in a previous study [7]. In this approach we studied the heating of Pd metal lines of different line widths. The temperature of the metal lines was characterized utilizing four-point probe measurements by studying the resistance versus power curves of the lines. After that, the heated metal lines were scanned using SThM. By correlating the SThM signal change at the heated lines with their temperature, we extracted a calibration factor as the slope of the linear relation. Following the principle of this approach, we obtained a Calibration Factor of 7.12 ± 0.52 mV/K to convert the electrical signal into a temperature change. Consequently, we can expect a relative error of around 7 % in the converted temperature maps. For the conversion, we then calculated the signal change of the heated map versus the unheated reference map and used the previously estimated calibration factor.

The reference maps display a standard deviation of 5-10 mV for the unheated case. Considering the calibration factor of 7.12 mV/K, we would expect a natural variation of around 1 K. Therefore, a temperature rise should exceed this value for reliable mapping. Based on the findings of our prior study we operate the SThM at a temperature increase of around 35 K, while applying a power of around 19 µW in the current study. It is noteworthy that the SThM can be operated at a temperature increase of 100 K and above. However, we choose to use lower power to prevent probe degradation or damage.

The lateral resolution of the temperature is linked to the thermal exchange radius of the tip. The thermal exchange radius describes the thermal exchange area between the probe and the sample as a disc shaped area [8]. Based on probe calibration, we considered a thermal exchange radius of around 200 nm, considering the utilized power configuration. Importantly, it is worth noting that the thermal exchange radius can vary with the power applied to the probe [7].

The temporal resolution of in-operando measurements with SThM probes correlates with the thermal time constant. For thermoresistive probes, the thermal time constant typically ranges in the order of a few hundred microseconds [9]. To validate that the SThM probe can resolve temperature changes during in-operando measurements, we maintained the electrical power applied for 0.8 s at each SThM probe measurement of the I-V sweep. By that means, the SThM probe has sufficient time to adapt to temperature changes. For probes with an even lower thermal time constant, lower hold times per measurement could be utilized. Additionally, if a higher temporal resolution is required, special SThM probes have shown to display thermal time constant in the microsecond regime [9].

Possible sources of errors in temperature quantification include thermal contact variations between the probe and sample, tip degradation or thermal drift. In order to reduce the impact of

the thermal contact, both the calibration and also the RRAM samples were coated with an Al_2O_3 capping layer, ensuring a comparable probe-sample thermal contact resistance in both cases. To address potential tip degradation, we recommend performing test scans on the calibration sample after frequent use of the same probe, to check if significant changes can be observed. Additionally, to minimize the impact of thermal drift, it is advisable to conduct a reference scan at zero device power after each measurement.

It is important to emphasize that the SThM measurements allow to report the temperature increase at the surface of the sample, induced by the heated conductive filament. The temperature increase of the buried filament is significantly higher, due to induced Joule self-heating which dissipates at the top electrode, the capping layer and the thermal contacts resulting in a reduction of the temperature at the surface. The simulation presented in this study allows us to estimate the actual temperature rise in the filament based on the measured temperature at the surface.

References

[1] D. Maldonado, S. Aldana, M.B. González, F. Jiménez-Molinos, F. Campabadal, J.B. Roldán, "Parameter extraction techniques for the analysis and modeling of resistive memories", Microelectronics Engineering, 265, 111876, 2022.

[2] M.J. Ibáñez, F. Jiménez-Molinos, J.B. Roldán, R. Yáñez, "Estimation of the reset voltage in Resistive RAMs using the Charge-Flux domain and a numerical method based on quasi-interpolation and discrete orthogonal polynomials", Mathematics and Computers in Simulation, 164, pp. 120-130, 2019.

[3] M.J. Ibáñez, D. Barrera, D. Maldonado, R. Yáñez, J. B. Roldan, "Non-Uniform Spline Quasi-Interpolation to Extract the Series Resistance in Resistive Switching Memristors for Compact Modeling Purposes", Mathematics, 9, 2159, 2021.

[4] D. Barrera, M.J. Ibáñez, F. Jiménez-Molinos, A.M. Roldán, J.B. Roldán, “A spline quasi-interpolation-based method to obtain the reset voltage in Resistive RAMs in the Charge-Flux domain”, Journal of Computational and Applied Mathematics, 354, pp. 326-333, 2019.

[5] M. A. Villena, M.B. González, F. Jiménez-Molinos, F. Campabadal, J.B. Roldán, J. Suñé, E. Romera, E. Miranda, “Simulation of thermal reset transitions in resistive switching memories including quantum effects”, Journal of Applied Physics, 115(21), 2014.

[6] T. Swoboda, X. Gao, C. M. M. Rosário, F. Hui, K. Zhu, Y. Yuan, S. Deshmukh, C. Köroglu, E. Pop, M. Lanza, H. Hilgenkamp, M. Muñoz-Rojo, “Spatially-Resolved Thermometry of Filamentary Nanoscale Hot Spots in TiO2 Resistive Random Access Memories to Address Device Variability”, ACS Applied Electronic Materials 5 (9), 5025-5031, 2023.

[7] T. Swoboda, N. Wainstein, C. Köroglu, X. Gao, M. Lanza, H. Hilgenkamp, E. Pop, E. Yalon, M. Muñoz-Rojo, “Nanoscale temperature sensing of electronic devices with calibrated scanning thermal microscopy”, Nanoscale,15, 7139-7146, 2023.

[8] S. Deshmukh, M. Muñoz-Rojo, E. Yalon, S. Vaziri, C. Köroglu, R. Islam, R.A. Iglesias, K. Saraswat, E. Pop, “Direct measurement of nanoscale filamentary hot spots in resistive memory devices”, Science Advances, 8, eabk1514, 2022.

[9] Y. Zhang, W. Zhu, F. Hui, M. Lanza, T. Borca-Tasciuc, M. Muñoz Rojo, “A Review on Principles and Applications of Scanning Thermal Microscopy (SThM)”, Advanced Functional Materials, 30, 1900892, 2020.
